# Dynamics of Existential Personality Fulfillment in the Course of Psychotherapy

**DOI:** 10.3390/bs10010021

**Published:** 2019-12-31

**Authors:** Marina M. Solobutina, Liliya R. Miyassarova

**Affiliations:** Institute of Psychology and Education, Kazan Federal University, 420008 Kazan City, Russia; miyalira@gmail.com

**Keywords:** existential psychotherapy, existential personality fulfillment, therapeutic changes

## Abstract

The purpose of the study is to explore the clients’ perceptions of therapeutic changes due to their existential fulfillment experience and authenticity in their relationships with the world. The content of the study reveals the subjective perceptions and experiences of clients about the changes in the understanding of themselves and the world in the course of existential psychotherapy. Consideration of the qualitative changes in a person’s life as a result of psychotherapy was based on the concept of existential fulfillment and de-sedimentation of “I-structure”. An opening up of opportunities for experiencing the fullness of human existence, as well as exploring ways of avoiding existential fulfillment, present themselves as key aspects in existential psychotherapy. Research methods are Existence Scale (A. Längle and C. Orgler); semantic differential for measuring therapeutic changes of clients in the course of existential psychotherapy; and factor analysis. Going through a psychotherapeutic experience has a positive effect on the dynamics of self-distancing indices, self-transcendence, freedom, and responsibility. The experience of existential personal fulfillment in psychotherapy leads to changes in human contact with oneself and the ability to successfully interact with the external environment. Experiencing the true existential level of living helps a person to be aware of their needs and to stay in contact with their feelings.

## 1. Introduction

In the practice of psychological counseling and psychotherapy, an intensive development of the culture of seeking psychological help is observed. People have a growing interest in exploring how they realize their life and the need for the improvement of its quality. There are renowned studies that assess the effectiveness of psychological assistance in various psychotherapeutic approaches. An important question is the choice of criteria for evaluating the effectiveness of psychotherapy [1]. The attention of researchers was mostly focused on the disappearance of manifestations of difficulties in a person’s life or painful symptoms, removal of psychological discomfort, or restoration of a sufficient level of functioning [2,3]. Nevertheless, the concept of therapeutic change is revealed through different dimensions in various therapeutic approaches. The analysis of therapeutic approaches to changes in the course of psychotherapy demonstrated the following disagreements regarding the nature, driving forces, and associated conditions of change.

Changes in the psychoanalytic approach occur in the course of self-inquiry and the actualization of traumatic children’s experience, when both are taking place in the safe conditions of psychotherapy. The results of successful psychotherapy are satisfaction from life, the ability to endure difficult emotions, building harmonious interpersonal and sexual relationships, and a greater understanding of personal needs. Gestalt therapy treats a person holistically, in conjunction with his/her past experience, attitudes, feelings, and limitations. Changes occur due to the return of spontaneity to the client while interacting with the environment. The therapist has no task to make changes in the client’s life; rather, the therapist helps clients to get into their own life. Therapy of a new solution involves stimulating the client to change. Changes occur when the client relives the painful situations of the past, can rethink them, and make new emotional and cognitive decisions. Psychotherapists within the cognitive direction believe that the condition of clients improves due to the regulation of emotions and changes to personal attitudes and life principles. The humanistic view of personality change lies in the achievement of a certain correspondence between the “I” and the real experience of a person. As a result of psychotherapy, clients become more open to experience, more realistic, and less protective. Personality changes are explained through the experience of feelings that were previously not banned from consciousness, and the inclusion of those in clients’ perceptions of themselves. Existential therapy initially sees life as a permanent change [4,5]. V. Frankl viewed therapeutic change as finding the global meaning of a situation or human life in general [6]. Psychotherapy involves revealing the clients’ ways of restraining themselves from the “fullness of existence” and the possibilities to make life choices consciously, based on the knowledge of their patterns [7,8]. Existential psychotherapist E. Spinelli explains therapeutic changes through the process of de-sedimentation [9]. If “I-structure” is open to external challenges, then some of its individual aspects are de-sedimented in a new way, like another structure. Each psychotherapeutic approach has its own ideas about the nature, driving forces, and associated conditions of therapeutic change [10], but it is common that changes in the course of psychotherapy are more related to the internal processes of the individual and do not always have a noticeable manifestation in the behavior or life events of the client.

A worldwide survey of counselors and psychotherapists’ perspectives on existential psychotherapy practices revealed the need to study the specifics of the existential psychotherapy process [11]. A meta-analysis of existential therapies pointed out their effectiveness in making meaning in life and reducing distress [12]. Existential psychotherapy needs to systematize and conceptualize its practice. There is a lack of empirical studies of the theoretical and methodological substantiation of the therapeutic process and the conceptual framework, the nature of interventions, and the essence of therapeutic change in the existential approach [13]. That inspired an area of research on the conditions for therapeutic personality and the evaluation of the therapy effectiveness [14,15,16]. C. Roger first formulated such conditions: Contact; client’s experience being in an incongruence state; therapist congruence; therapist unconditional positive regard; therapist empathy; and empathic and unconditional communication with a client. Humanistic and existential practices were revealed through therapists’ skills, phenomenological method, and relational perspectives. Existential therapeutic practices emphasize the importance of subjectivity and the uniqueness of human experience. To conceptualize the practice of existential psychotherapy, it is important to study the clients’ representations and assessments about their therapeutic experience.

The study of the clients’ personal meanings, immersion in their everyday life, awareness of their capabilities and limitations, and clarification of attitudes toward life challenges and dilemmas are the main aspects of the therapeutic work that helps people achieve fullness of existence. Therapeutic changes in existential psychotherapy are understood as changes in clients’ personal meanings. In order to understand the nature, driving forces, and conditions of clients’ personal changes, it is necessary to investigate the clients’ subjective perceptions about psychotherapy. This article presents the results of a study of clients’ therapeutic changes in existential psychotherapy.

## 2. Materials and Methods

The research sample: Psychotherapists who identified their practice as existential and had specific institutional training were asked to provide their clients with therapeutic experience of over 50 h, in order to take part in the investigation. The sample was selected by inclusion criteria (existential therapeutic practice, sustainable therapy, and adults). The sample involved 40 participants aged 21–53 years who underwent the experience of personal psychotherapy. It comprised 30 women and 10 men. The average age of the subjects was 35 years. The average time of the psychotherapy was 95 h.

The type of research utilized a mixed approach: quantitative and qualitative. This article contains the results of the first quantitative stage. At the second stage, a phenomenological interview was conducted with clients of psychotherapy (the Colazzi method). The results of the phenomenological research will be covered in other publications. Ethical standards were observed during the study.

To study the ideas of clients about therapeutic changes, we relied on the methodology of existential psychotherapists A. Längle [17] and E. Spinelli [18]. The purpose of the study was to explore the clients’ perceptions of therapeutic changes due to their existential fulfillment experience and authenticity in their relationships with the world. Research hypotheses: (1) Therapeutic personality changes are associated with the process of de-sedimentation of the “I-structure”, when the psychotherapy clients change their attitude to their past and present, reconnect with their feelings and values, and gain the habit of healthy contact with the environment; (2) the driving forces of the de-sedimentation of the “I-structure” in the process of psychotherapy are the experience of existential fulfillment, the unfolding and full living of stopped or previously forbidden feelings, detection and appropriation of the previously torn realities of themselves, and building harmonious interpersonal relationships.

To test the hypotheses of the study, the research methods used were as follows: Existence Scale (A. Längle and C. Orgler); semantic differential for measuring therapeutic changes of clients in the course of existential psychotherapy; and factor analysis and *t*-test.

The Existence Scale measures existential fulfillment as it is perceived subjectively by the subject. The level of existential fulfillment depends on the meaningfulness of life, internal harmony, and the conformity of decisions and actions. The test is based on Frankl’s existential–analytical theory and Längle’s four fundamental motivation concepts. It consists of 46 items and include subscales: self-distance, self-transcendence, freedom, responsibility, and personality. The existential fulfillment indicator is the total of intermediate indicators for four subscales.

Semantic differential is a type of rating scale designed to measure the connotative meaning of objects, events, and concepts. The connotations are used to derive the attitude toward the given statements. We used a semantic differential to measure therapeutic changes of clients in the course of existential psychotherapy toward the concept of existential fulfillment by A. Längle and de-sedimentation of “I-structure” by E. Spinelli. The results were processed by factor analysis. The method belongs to the methods of experimental semantics which speak to the construction of semantic spaces (Ch. Osgood). It is widely used in the research studies related to the perception and behavior of a person since it analyses attitudes and personal meanings. A list of 60 statements about therapeutic changes was made to identify the factors of de-sedimentation of the person’s “I-structure” in the course of psychotherapy.

The subjects assessed themselves and their lives in different aspects, which are divided into two columns: before psychotherapy and after psychotherapy. The column “before psychotherapy” reflected the memories of the subjects about themselves and their lives. The choice of this research strategy is based on the research of C. Rogers on the measurement of the client’s personal changes [19]. C. Rogers showed by experiments that clients’ memories of themselves reflect the situation more objectively than their real answers in the past. He attributed this to the diminishing influence of psychological defense as a result of psychotherapy and the emergence of the ability to give more accurate self-esteem.

## 3. Results

### 3.1. The Experience of Existential Fulfillment in the Course of Psychotherapy

Significant differences in the indicators of existential fulfillment of the subjects before and after psychotherapy were revealed (*p* ≤ 0.01). Getting a psychotherapeutic experience has a positive effect, and the level of existential fulfillment increases (Table 1). The average marks on the subscales of self-distance (SD), self-transcendence (ST), freedom (F), responsibility (V), and personality (P) after long-term psychotherapy are significantly higher in comparison with the subjects’ evaluation before the psychotherapy. The total indicator of existential fulfillment (E-G) is higher after the psychotherapy (*p* ≤ 0.01).

The subjective perception of the subjects about their existential fulfillment based on the psychotherapeutic experience is as follows. An increase in the self-distance scale was found. This result indicates the development of the ability of the subjects to show self-reflection and make judgments about their own behavior and emotional state. The subjects believe that they have improved their ability to distance themselves from their short-term desires in favor of something more important. More flexibility in the distribution of life contexts is seen in the continuum of what is considered important or unimportant. In addition to self-reflection, this scale also demonstrates a person’s ability to de-reflection. Personal desires and immediate needs were given priority and required quick implementation before the psychotherapy. There occurred the expansion of contexts in the course of psychotherapy: The situation could be of interest, even if it did not quite correspond to the immediate needs of the person. More freedom to choose between “I want” and “I have” to was observed. The subjects noted that the focus on themselves and their concerns had decreased. They began to notice other people more and take their needs into account. The ability to see different possibilities for problem-solving improved. Resistance to stress increased. The perception of the world became more multifaceted.

Therapeutic changes in terms of freedom and personality were revealed. The subjects noted that the value of life increased. The attitude toward life became more positive. Psychotherapy clients gave the challenges in significant areas a new meaning. Their contact with themselves improved. The participants began to better understand the meaning of what is happening in life. Solitude was no longer perceived as something wrong. At the same time, they started to express increased empathy toward others. Satisfaction from living life improved. Thoughts of deprivation due to unfulfilled desires stopped bothering them. The subjects built awareness by defining the limits of their responsibility. They began to like to listen to themselves, to form their own opinion based on their personal meanings.

Changes in releasing feelings were noticed. They began to pay more attention to feelings as indicators of one’s needs. It became easier for the subjects under consideration to make decisions, even the ones related to the articulation of unpleasant feelings. Instead of being acted upon, clients started acting themselves and showing the ability to choose. They began to better navigate the situation and choose their behavior depending on a more realistic understanding of the situation and personal abilities/limitations. The clients began to experience more inner freedom and independence. More freedom from other people’s expectations was evidenced, as well. Self-confidence increased.

The subjects began to better assess the feasibility of their decisions, as well as to predict the consequences of their actions. Understanding what is important to them allowed them to gradually abandon multitasking. Focusing on the most important and necessary things that influenced their perception of everyday life and their well-being improved. The subjects began to feel less tired and anxious, they became more concentrated and attentive, and they began to feel and distribute their strength better. The understanding of the limits of personal responsibility changed: The area of responsibility became more realistic.

### 3.2. Working Out a Semantic Differential to Study the Participants’ Ideas of Therapeutic Changes

We based our study on the theory of E. Spinelli, who explains therapeutic changes through the process of de-sedimentation. If “I-structure” is open to external challenges, then some of its individual aspects are de-sedimented in a new structure. The selected characteristics of identity were transformed into a semantic differential, which included 60 statements. The subjects were asked to evaluate each of the characteristics as inherent in them and not, before and after the psychotherapy. At the next stage of this study, we compiled a summary protocol of the values of the semantic differential. The raw values were processed by using factor analysis. That allowed us to present all the characteristics in a four-dimensional space. We identified four factors that reflected the therapeutic changes through the process of de-sedimentation: the attitude to the past, contact with feelings and body, relationship with the world, and values and personal meanings. The activity of the subjects was assessed by summing up the scores and identifying the average score for each subject individually. Based on the results of factor analysis, we divided all the presented qualities into four factors. The characteristics with a small value of the correlation coefficient (<0.3) were excluded from the analysis. Factor analyses were conducted on nonredundant questionnaire items (KMO = 0.75; Bartlett’s test, *p* < 0.001). The eigenvalue = 1 criterion was used and resulted in four factors; the scree plot indicated a four-factor solution. We used a varimax rotation for simple structure; the four-factor solution was interpretable.

The first factor is revealed by the statements connected with the understanding of the influence of the past on the present (Table 2). The inverse nature of the correlation between the statement and the factor is observed for such characteristics as openness to changes in one’s life, forgiveness of past offenses, and acceptance of one’s past. We called the first factor “Attitude to the Past”, in compliance with the factor values.

The second factor is revealed by the statements connected with feelings and body sensations (Table 3). The psychotherapeutic process allows individuals to restore bodily sensitivity and openness to feelings and to understand their condition and needs. The inverse nature of the correlation between the statements and the factor is observed for such characteristics as awareness of feelings, acceptance of the human nature ambivalence, vitality, and contact with bodily sensations. We called the second factor “Contact with Feelings and Body”, in compliance with the factor values.

The third factor is the most powerful and thorough. It is revealed by the statements connected with personal attitude to the environment and social integration (Table 4). The perception of and relationships with the world affect the quality of the individual’s life. The inverse nature of the correlation between the statements and the factor is observed for such characteristics as opportunities for self-expression, tolerance for uncertainty of the world, openness to contact with the world, taking responsibility for life events, self-knowledge, and development through relationships with other people. We called the third factor “Relationship with the World”, in compliance with the factor values.

The fourth factor is revealed by the statements connected with awareness of values in the course of psychotherapy (Table 5). Existential psychotherapy focuses on the issues of the meaning-making, feeling of awareness in one’s everyday life, and determining personality support that helps withstand the uncertainty of life. The inverse nature of the correlation between the statements and the factor is observed for such characteristics as awareness of the meanings of events, fullness of life, loss/discovery of the meaning of life, and attitude to changes in life. We called the fourth factor “Values and Personal Meanings”, in compliance with the factor values.

Significant differences in indicators of the subjects “I-structure” before and after the psychotherapy were revealed (*p* ≤ 0.01). Getting a psychotherapeutic experience has a positive effect on identified factors (Table 6). The average value on the factors that reflect the therapeutic changes through the process of de-sedimentation (attitude to the past, contact with feelings and the body, relationship with the world, and values and personal meanings) after a long-term psychotherapy are significantly higher in comparison with the subjects’ evaluation before psychotherapy.

## 4. Discussion

The results of the study led to the conclusion that the psychotherapeutic experience has a positive effect on the de-sedimentation of the “I-structure” of the personality and the change in the experience of existential fulfillment. Psychotherapy contributed to the development of the ability of the subjects to form self-reflection and make judgments about their own behavior and emotional state. The possibility of inner distance of the individuals to themselves is realized by means of psychotherapeutic research. Such a distancing experience helps to develop clients’ reflection. It was revealed that changes occurred in individual methods of adaptation after a sustainable personal therapy, i.e., the shift from distortions in self-perception and the perception of others to the awareness of what is happening in the internal and external reality of a person.

The clarification of the values of the individual in the course of psychotherapy is carried out through the acceptance of the emotional side of the inner life. The subjects noted that, on the one hand, they improved their ability to distance themselves from their feelings, desires, and prejudices, in order to realistically assess the situation. On the other hand, they recognized an improved contact with their feelings and recognition of their needs. It became easier to make decisions, even those related to unpleasant feelings. Instead of being acted upon, clients start acting themselves and showing the ability to choose. A psychotherapeutic study of clients’ attitudes to life challenges and their ways to solve dilemmas allowed them to understand their ways of living. Changes that are inherent in life in general, in the course of psychotherapy, are associated with understanding the difficulties in human life. The safe space of psychotherapy revealed the possibility of detecting the meaning of anxiety when meeting with new experiences and uncertainties. As a result of psychotherapy, there was more freedom in the authentic way of life, while achieving harmonious relationships with other people. The processes of individuation and social integration have become more balanced and conscious. The subjects felt that, with the help of psychotherapy, they became more open in their perception of themselves and the world. They believed that the study of one’s own life, feelings, and ways of interacting with the social reality made it possible to better navigate the world and see opportunities in life’s challenges. Life became more filled with essential things.

In existential psychotherapy, the idea of the past is not considered in isolation from the client’s present. The past is as important as the present. People’s past painful experiences often prompt them to seek psychotherapeutic help. The psychotherapeutic process permits them to respond to delayed feelings and explore and change the attitude to the situation. After psychotherapy, memories of past events no longer caused strong feelings. The subjects managed to overcome their grievances about other people and stop blaming others. The desire to change their past lost relevance. They took the past as part of their life. The actualization of deferred feelings allowed them not to avoid unpleasant experiences in the present. The subjects became tolerant of disturbing situations. Changing the focus of attention from the past to the present and the future allowed the subjects to become more open to changes in life. The subjects began to make decisions based not only on cognitive understanding but also based on emotional and bodily experience. The ability to contain unpleasant feelings increased. There was an acceptance of the ambivalence of human nature. The therapeutic experience increased tolerance for uncertainty, anxiety, and risk, and, as a result, made them open to contact with the world and changes in life. Relationships with other people began to be perceived as enriching.

## 5. Conclusions

Personal psychotherapy helps to address issues of existential fulfillment, which in turn leads to therapeutic changes. Therapeutic personality changes are associated with the process of de-sedimentation of the “I-structure”, when psychotherapy clients change their attitude to their past and present, reconnect with their feelings and values, and gain the habit of healthy contact with the ambient environment. The driving forces of the de-sedimentation of the “I-structure” in the course of psychotherapy are the experience of existential fulfillment; the unfolding and living through the stopped or previously forbidden feelings; detection and appropriation of the previously torn self-identities; and building harmonious interpersonal relationships. In existential psychotherapy, the experience of a person’s involvement in life assumes central importance. The experience of long-term personal therapy helped clients to determine their “existential place in the world”, to take responsibility for their life events, and to withstand stress in difficult situations.

The identified therapeutic changes in clients of a sustainable personal existential psychotherapy included the following:

- Ability to stabilize interpersonal relationships;
- Willingness to meet with internal and external requirements and challenges in accordance with their own values;
- Internal openness to the requests and suggestions of the world;
- Internal consistency with the chosen lifestyle;
- Responsibility for making decisions even in a state of tension and anxiety;
- In general, life is filled with meanings and inner satisfaction.

## Figures and Tables

**Table 1:** The *t*-test on indicators of subjects’ existential fulfillment.

*t*-Test	SD	ST	F	V	P	E	G
mean before psychotherapy	28	52	34	48	80	82	162
mean after psychotherapy	32	75	47	54	106	101	208
*t* (*p ≤* 0.01)	7.28	7.33	7.54	7.27	7.55	7.52	7.72

**Table 2:** Factor “Attitude to the Past”.

Statements	Factor Value
I am open and not afraid of changes in my life	0.57
I do not feel strong emotions when recalling past events	0.49
I can forgive close ones for their wrong behavior in the past	0.34
I treat my past with acceptance as part of my life	0.30
I avoid disturbing situations and anxiety	−0.31
I want to change my past	−0.41
I tend to blame others for my troubles	−0.45
I try to distract myself when I have unpleasant feelings	−0.48
There is an imbalance of my presence in different areas of my life	−0.49
I have a strong fear of my own death and the death of loved ones	−0.60
I get very upset when I recall some events from my past	−0.71

**Table 3:** Factor “Contact with Feelings and Body”.

Statements	Factor Value
I am well aware of my feelings and the reasons for their occurrence	0.59
I accept the inconsistency and duality of my nature	0.59
My way of life is conscious	0.55
I understand and feel what it means to be alive	0.53
I understand my condition and body language	0.48
My body is a source of pleasure and resources	0.47
I am aware and take into account all my experience in making decisions (cognitive, emotional, and physical)	0.47
I feel my whole body and its borders well	0.44
I can be alone and not avoid solitude	0.30
I find it difficult to concentrate and understand bodily sensations	−0.30
Sometimes I do not understand what is happening to me and the causes of my condition	−0.32
I do not feel alive	−0.35
I can’t live meaningfully	−0.42
I find it difficult to withstand my inconsistency and duality	−0.51

**Table 4:** Factor “Relationship with the World”.

Statements	Factor Value
I do not feel a lack of self-expression	0.80
My self-expression is filled with authenticity	0.61
I accept the inconsistency and duality of my nature	0.59
I am open and not afraid of changes in my life	0.57
I can handle an anxiety	0.48
There is an imbalance of my presence in different areas of my life	0.48
I am open and not afraid of indeterminacy of life	0.45
I am open to contact with the world	0.43
I am responsible for all life events	0.42
I accept various sides of my personality	0.38
Relationships with people help me to understand myself better and develop	0.32
I fear that other people can destroy my worldview and self-image	−0.30
I tend to blame others for my troubles	−0.48
I try to fit the ideal image when communicating with people	−0.48
I try not to make risk-based decisions	−0.49
I find it difficult to withstand my inconsistency and duality	−0.51
I am lost and afraid to take any action in situations of uncertainty	−72
I find it difficult to express myself	−0.80

**Table 5:** Factor “Values and Personal Meanings”.

Statements	Factor Value
I feel the fullness of my life	0.80
I accept the inconsistency and duality of my nature	0.51
I afford a senseless pastime	0.51
I am open and not afraid of indeterminacy of life	0.49
I find valuable in every day	0.48
I do not avoid unpleasant feelings	0.46
I am aware and take into account all my experience in making decisions (cognitive, emotional, and physical)	0.39
The events of my past are meaningful and valuable	0.32
I can’t live meaningfully	−0.30
I do not feel alive	−0.30
I cannot stay long alone	−0.30
My life becomes filled only when I complement someone	−0.32
I close when interacting with other people	−0.37
I don’t accept some sides of my personality	−0.45
I feel the loss of the meaning of life	−0.49
I blame some people for their behavior	−0.60
I try to keep my lifestyle, and I don’t like changes	−0.80

**Table 6:** The *t*-test on indicators of de-sedimentation of “I-structure”.

*t*-Test	Attitude to the Past	Contact with Feelings and Body	Relationship with the World	Values and Personal Meanings
mean before psychotherapy	3	3	3	4
mean after psychotherapy	5	6	5	5
*t* (*p* ≤ 0.01)	6.35	6.52	6.72	6.32

## Data Availability

The datasets used and analyzed during the current study are available from the corresponding author, upon reasonable request.
